# Dual Sensory Loss, Mental Health, and Wellbeing of Older Adults Living in China

**DOI:** 10.3389/fpubh.2019.00092

**Published:** 2019-04-24

**Authors:** Chyrisse Heine, Cathy Honge Gong, Colette Browning

**Affiliations:** ^1^Department of Community and Clinical Allied Health, La Trobe University, Bundoora, VIC, Australia; ^2^Centre for Research on Ageing, Health and Wellbeing, Research School of Population Health, College of Health and Medicine, The Australian National University, Canberra, ACT, Australia; ^3^International Institute for Primary Health Care Research, Shenzhen, China

**Keywords:** sensory loss, mental health, depression, wellbeing, life satisfaction, dual sensory loss, China

## Abstract

**Introduction:** Deterioration in vision and hearing commonly occurs as adults age. Existing literature shows that Dual Sensory Loss (DSL) is a prevalent condition amongst older adults. In China, it has been estimated that 57.2% of the population experience DSL. Based on a small number of research papers, it has been identified that DSL influences mental health and wellbeing. The aims of this study were to explore the relationship between DSL and mental health and wellbeing in a sample of older adults residing in China; and investigate whether the comorbidities of functional dependency [Activities of Daily Living (ADL) and Instrumental Activities of Daily Living (IADL)] and chronic diseases influence the impacts of DSL on mental health and wellbeing.

**Method:** The China Health and Retirement Longitudinal Study Wave 2, 2013 data collection of a sample of people aged 60 years and over (*n* = 8,268) was used in this study. The sensory loss variables selected for analysis included a combined variable of self-reported vision and hearing loss (DSL). Mental health was measured by depression, and general wellbeing was measured by life satisfaction. In addition, chronic diseases, and limitations in IADL and ADL were used to test how their comorbidities with DSL influence mental health and wellbeing. Results were analyzed descriptively and using regression and modeling techniques.

**Results and Discussion:** DSL was significantly and positively associated with advanced age, having difficulty in any ADL or IADL and experiencing depression and less life satisfaction. The observed negative associations between DSL and mental health or wellbeing, are indirect and could be partially explained by its comorbidity with chronic diseases and relationship to functional limitations. It is recommended that health services in China screen for DSL in older people and develop integrated services to assist with appropriate management and rehabilitation of older people with DSL focusing on both functional and mental health issues.

## Introduction

Many adults experience sensory losses as they get older (1, 2). Dual Sensory Loss (DSL) is defined as “the acquired loss, in various degrees of severity of both vision and hearing acuity, associated with aging and prevalent in older adults” (3) (p. 1). The prevalence of vision loss and hearing loss in older adults is high. In a population study of adults aged 75 years and over (*n* = 14,600), 12.4% were visually impaired, 10.3% had low vision and 2.1% were blind (4). Likewise, in the Beaver Dam, Wisconsin study (5), the prevalence of hearing loss in older adults (average age of 65.8 years) was high (45.9%). The prevalence of DSL reported in the literature ranges from 5.9% in adults aged over 50 years (6) to as much as 25% in adults aged over 80 years (7). The prevalence of vision and/or hearing loss in older adults is also high in China. In a recent publication on this topic, Heine et al. (8) concluded that vision loss was present in 80.2% of the sample, 64.9% of the sample reported hearing loss and 57.2% experienced DSL. In comparison to the high prevalence rate of these sensory losses, there was low usage of aids (10% of respondents wore glasses regularly and only 0.8% used hearing aids) resulting in a high level of unmet needs for glasses (54.9%) and hearing aids (63.9%).

The causes of acquired vision and/or hearing loss in older adults are varied. Common causes of vision loss in older adults include age-related macular degeneration, glaucoma, ocular complications of diabetes mellitus and age-related cataracts (1, 9, 10). Common causes of hearing loss in older adults include presbycusis, noise-induced hearing loss, cerumen occlusion, middle ear ossification, viruses, bacteria, heart condition, stroke, brain injury, tumor, or ototoxic medication (11, 12).

The sensory losses resulting from vision and hearing acuity changes are numerous. People with vision loss experience vision ranging from moderate visual impairment to severe visual impairment to blindness (13). People with vision loss often have difficulty seeing fine detail, visualizing distant objects, discriminating detail, and reading print. In addition, they may have poor contrast sensitivity, intolerance for glare and restricted mobility. In China, poor vision for long distance is found to be one of the major reasons for vision impairment or blindness in later life (10). Similarly, hearing loss ranges from mild to profound loss with consequent impairment typically including varying degrees of difficulty with: hearing sounds, speech discrimination, and perception in quiet and noisy environments, or environments that are echoic and difficulty processing information (14). DSL thus results in a combination of these sensory difficulties. Brabyn et al. (15) suggested that the combined effect of vision and hearing loss could cause complex interactions with task performance because of the dimensions of both types of deficits.

DSL is also associated with poorer health, decreased wellbeing and quality of life. In a systematic review of the literature, Heine and Browning (16) reviewed 42 studies investigating DSL and its comorbidities. Results of this review found that the most common association examined in the literature was between DSL and functional difficulties [such as difficulty completing Activities of Daily Living (ADLs) and/or Instrumental Activities of Daily Living (IADLs)]. For example, based on the Longitudinal Study on Aging, Brennan et al. (17) explored the functional abilities of 5,151 adults aged 70 and older and found that one fifth of older adults with DSL had associated difficulty with ADLs and IADLs as compared to those with a single sensory loss. Outcomes of this study and a further study in China (10) suggested that effective vision and hearing rehabilitation (including sensory resources or aids) are essential for those with DSL to maintain their functional independence and quality of life. In a study of 393 members of the Hong Kong Chinese community aged 60 years and older, Chou (18) concluded that everyday competence was significantly and negatively associated with depressive symptoms in people with low functional capacities.

Within the DSL literature, studies investigating the association between DSL and mental health are rare. Based on the outcomes of a systematic review conducted by Heine and Browning (19), only a few studies have investigated the relationship between DSL and depression, whilst even fewer have investigated the association between DSL and quality of life. There were no studies in this systematic review that investigated the association between DSL and anxiety. According to Chou and Chi (20), in a sample of 2,003 community dwelling Chinese residents residing in Hong Kong, 6.5% displayed depression, with vision impairment significantly related to depression whilst hearing impairment had no significant association with depression. Similarly, based on a large population study (*n* = 2,689), McDonnall (21) found a significant relationship between DSL and depression, particularly at the onset of DSL. Poor Quality of Life has also been significantly associated with DSL (22), although the evidence base is small.

Since the review on DSL and mental health was published by Heine and Browning (19), some new studies have appeared. For example, based on cross-sectional data of 8,500 adults aged 50 years and older from Wave 1 of the Irish Longitudinal Study on Aging (TILDA), collected between 2009 and 2011, Lehane et al. (23) found that the loss of one's own or one's spouse's hearing and/or vision is a stressful experience. Furthermore, individuals with DSL reported greater levels of both anxiety and depression, whilst their spouses reported greater levels of depression only when compared to couples without sensory loss. Recent studies of Asian populations have shown similar impacts of sensory loss on mental health. Based on the data of 5,832 individuals from the 2006 to 2014 Korean Longitudinal Study of Aging (KLSoA), Han et al. (24) found that hearing, vision and dual sensory impairment are significantly associated with depressive symptoms. These authors suggested that treatment or rehabilitation of either hearing or vision impairment would assist in the prevention of depression.

Heine and Browning (16) conducted a systematic review of DSL and other comorbidities such as life satisfaction, chronic illnesses, and dementia. Again, there were few studies investigating these relationships. For example, Bazargan et al. (25) explored the psychological wellbeing of 988 older African American people and found that those with DSL experienced multiple comorbidities such as poorer health and disparities in their health and social roles as compared to people with no DSL. In a study based on the China Health and Retirement Longitudinal Study (CHARLS) data of 8,268 older adults aged 60 years and over residing in China (published in this Special Topic), Heine et al. (8) found that over two-thirds of respondents reported vision loss (80.2%) and hearing loss (64.9%) whilst over a half of respondents (57.2%) reported DSL. Furthermore, vision and hearing loss together with the unmet needs for glasses and hearing aids were negatively associated with participation in social activities.

There is thus limited information about DSL and its association with mental health and wellbeing and even less information about these associations in the Chinese older population^1^. The prevalence of DSL will increase as China's population ages. It is important to understand how DSL impacts on mental health and wellbeing and how other age-related conditions may impact on that relationship. This information can help inform the design of health services and rehabilitation programs to improve the quality of life of older Chinese with DSL by adding valuable life to years (26).

The aims of this study are to:

1. Explore the relationship between DSL and mental health and wellbeing (as measured by depressive symptoms and life satisfaction).
2. Investigate whether the comorbidities of functional dependency (ADL and IADL) and chronic diseases influence the impacts of DSL on mental health and wellbeing.

## Methods

### Data Source

In the current study, the China Health and Retirement Longitudinal Study (CHARLS) dataset was used. The CHARLS is a national, longitudinal survey of people aged 45 years and over that explores aging and health in China (27). Detailed information on demographic characteristics, social and economic conditions, functional impairments, chronic diseases, activities of daily living (ADL) limitations, disabilities, and psychological wellbeing were collected via face-to-face interviews in respondents' homes. In the first wave of CHARLS (in 2011), random selection of participants took place using a multi-stage probability-proportional-to-size technique, stratified by regions and then by urban districts or rural counties and per capita gross domestic product (GDP). In the second wave of data collection (in 2013), respondents aged 45 and over were surveyed (*n* = 18,246), in which 14,988 were from Wave 1. Missing values had not been imputed in the survey data before its availability to the public. Individual weights denoting the inverse of the probability that the observation are included because the sampling design with household and individual non-response adjustments, are used for analysis. In the current study, only older adults (those aged 60 years and over) were included for analysis (*n* = 8,268). CHARLS was approved and is overseen by the Biomedical Ethics Review Committee of Peking University. There is no need to have a further ethics approval for the secondary data analysis. The software used for the data analysis in this study is STATA 15.1.

### Measures

In the CHARLS dataset, there are multiple questions asking respondents about their vision and hearing. To form the construct of DSL, two questions relating to vision were included: (1) How good is your vision for seeing things at a distance, like recognizing a friend from across the street (with glasses or corrective lenses if you wear them, 1 = excellent, 2 = very good, 3 = good, 4 = fair, or 5 = poor)? (2) How good is your vision for seeing things up close, like reading ordinary newspaper print (with glasses or corrective lenses if you wear them, 1 = excellent, 2 = very good, 3 = good, 4 = fair, or 5 = poor)? A fair/ poor answer to either or both of these questions was classified as vision loss. One question relating to hearing was used for measuring hearing status: “Is your hearing excellent, very good, good, fair, poor, (with a hearing aid if you normally use it and without if you normally don't)?” A fair/poor answer to this question was classified as hearing loss. The variable of DSL was defined as having both fair/poor vision and fair/poor hearing.

Demographic variables included age, gender, marital status, and ethnicity (whether the minority or majority). Educational attainment, relative living standard, and residence (urban or rural) were used to measure Socio-Economic Status (SES), which have impacts on the access to health services use and other social and economic resources (28–30), hence may change the impacts of DSL on depression and wellbeing. Urban and rural areas are defined according to the most recently published statistical standard by the National Bureau of Statistics (NBS) (31), where urban areas include both communities and villages located within access to the city or town facilities, while rural areas include only villages out of the city or town facilities (32). Educational attainment was categorized in three groups: (1) Primary schooling or under (illiterate or some primary schooling); (2) Second schooling (completed primary or secondary school); (3) college or above degrees.

In order to address the impact of the large variations across regions in both income and living costs, the relative living standard (instead of absolute income or expenditure) was used in this study to measure SES (32). The relative living standard is self-reported by respondents by answering the question: “Compared to the average living standard of people in your city or county, how would you rate your standard of living relative to those in your city/county: much better, a little better, about the same, a little worse, much worse?” Answers were grouped into three categories: “better”; “worse” and “about the same,” since using five groups did not enhance the interpretation beyond using the three categories used in the model.

The comorbidities of DSL included chronic diseases, ADL and Instrumental Activities of Daily Living (IADL). Chronic diseases were measured by whether the participant had any diagnosed conditions (hypertension, an unusual (high or low) lipoprotein level, diabetes, cancer, chronic lung diseases, liver or gallbladder disease, heart disease, stroke, kidney disease, stomach, or other digestive disease, emotional, nervous, or psychiatric problems, memory-related disease, rheumatism/arthritis or asthma). ADL limitations were measured by any self-reported difficulty in any of the following activities of daily living domains: bathing /showering, eating, dressing, getting into or out of bed, using the toilet, or controlling urination and defecation. IADL limitations were measured by any self-reported difficulty in any of the household activities: such as doing household chores, preparing hot meals, shopping for groceries, managing your money and taking medications.

The mental health and general wellbeing outcomes were measured by two variables: (1) depressed or not and; (2) life satisfaction. Depression in this study was measured by a group of ten questions in CHARLS selected from the Center for Epidemiologic Studies Depression Scale (CES-D) such as: being bothered by things, having trouble concentrating on things, feeling depressed, feeling everything was an effort, not feeling hopeful about the future, feeling fearful, having restless sleep, not being happy, feeling lonely or could not get going. A score of zero to three was assigned to each answer and then summed and divided into two groups according to the distribution of scores: no depression (score of 0–15), mild/ moderate/ major depression (score of 15–30). Life satisfaction was measured according to the respondent's response to the question “Please think about your life-as-a-whole. How satisfied are you with it? Are you completely satisfied (= 5), very satisfied (= 4), somewhat satisfied (= 3), not very satisfied (= 2), or not at all satisfied (= 1)?” Satisfaction is defined as satisfied (= 5), very satisfied (= 4), somewhat satisfied (= 3), while dissatisfaction is defined as not very satisfied (= 2), or not at all satisfied (= 1).

### Methods

Descriptive analyses of DSL prevalence rate by age, gender, education and relatively living standard are first reported. These data were followed by Spearman correlations between the prevalence of DSL and the physical and mental health measures as well as the general wellbeing measure. Finally, multivariate logistic regression was used to investigate the associations between DSL and mental health or wellbeing by controlling for all the predisposing factors (age, gender, urban/rural residence, marital status, ethnic group), SES (education and relative living standard), functional dependency and chronic diseases.

As a *P* < 0.05 has been widely used for significance tests in health literature, we keep both the actual *p*-values (in the last table of multivariate regression results) and the indication of significance level (^***^P < 0.01, and ^**^P < 0.05 in all tables).

## Results

The prevalence rate of DSL was obtained and analyzed by age and gender (see Table 1) and education, urban/rural residence and SES (relative living standard) (see Table 2).

**Table 1:** Prevalence of DSL by age and gender among older Chinese, 2013.

**Age group**	**Total sample size CHARLS 2013**	**Persons with DSL**	**Men with DSL**	**Women with DSL**
60–64	2,677	55.2	53.8	56.5
65–69	2,197	56.6	55.2	58.1
70–74	1,539	60.0	62.5	57.4
75+	1,855	58.5	57.5	59.6
Total 60+	8,268	57.2	56.7	57.8

*Sample size and weighted proportions from CHARLS 2013. http://charls.pku.edu.cn/en*.

**Table 2:** Prevalence of DSL by education, rurality, and SES among older Chinese, 2013.

**Persons**	**Sample size CHARLS 2013**	**Dual poor/fair vision & hearing**
Total 60+	8,268	57.2
Primary schooling or under	4,768	59.1
Second schooling	3,343	55.2
College and above	155	42.6
Rural residence	4,957	61.0
Urban residence	3,311	52.1
Better living standard	256	47.2
Average living standard	1,674	53.2
Worse living standard	4,151	59.8

*Sample size and weighted proportions from CHARLS 2013. http://charls.pku.edu.cn/en*.

As can be seen from Table 1, just over half of all participants reported DSL (57.2%). The prevalence increased across the age groups until 74 years with a slight decrease in prevalence in those aged 75 years and over. The pattern was somewhat different between men and women: for men, those aged 70–74 years had the significant and highest prevalence of DSL (62.5%), while for women, those aged 75 years and over had the highest prevalence of DSL (59.6%) but it is statistically insignificant.

Table 2 shows that those with a lower attainment in education (with primary school or under, or second schooling) reported a significantly higher prevalence rate of DSL (59.1 and 55.2% respectively), whilst those with the highest level of education (college and above) reported the lowest prevalence of DSL (42.6%). Those respondents living rurally reported a significantly higher prevalence of DSL (61.0%) compared to respondents living in an urban environment (52.1%). The prevalence of DSL was 47.2% for older people with a relatively better living standard, and increased to a significantly higher rate (59.8%) for those with relatively worse living standard.

Table 3 shows the relationship between DSL and depression, life dissatisfaction, any ADL or IADL limitation, and any chronic disease. Respondents with DSL had a higher proportion of reporting depression (18.1%), life dissatisfaction (14.58%), any chronic disease (76.72%) and difficulty in completing ADLs (11.21%) and IADLs (23.28%), compared to the group with no DSL. In addition, there are significant comorbidities between DSL and chronic diseases, ADLs or IADLs. About 44.95, 6.77, and 13.03% had DSL together with any chronic disease, ADL or IADL, respectively.

**Table 3:** The relationship between of DSL and mental health, wellbeing, functional limitations, and chronic disease.

**Proportions (%) among those aged 60+**	**Depression**	**Life dissatisfaction**	**Any ADL limitation**	**Any IADL limitation**	**Any chronic disease**
ALL	14.06	11.95	13.72	23.06	73.34
DSL_No	8.88	8.45	8.41	14	68.55
DSL_Yes	18.1^*^	14.58^*^	11.21^*^	23.28^*^	76.72^*^

* *Indicates significant differences between DSL and non-DSL group at a significance level of 5%*.

*CHARLS 2013. http://charls.pku.edu.cn/en*.

Spearman correlations were also calculated for the relationship between DSL and depression, life dissatisfaction, any ADL limitation, any IADL limitation and any chronic diseases (see Table 4). The Spearman correlations in Table 4 indicate that depression, life dissatisfaction, ADL limitation, IADL limitation, or having any chronic disease are all significantly and positively correlated with DSL.

**Table 4:** Spearman correlations between key variables.

**Spearman correlations**	**DSL**
Depression	0.112^*^
Satisfaction	−0.077^*^
Any ADL limitation	0.042^*^
Any IADL limitation	0.098^*^
Any chronic disease	0.091^*^

* *Indicates significant correlation with DSL at a significance level of 5%*.

*CHARLS 2013. http://charls.pku.edu.cn/en*.

Table 5 presents results from the multivariate logistic regression models to investigate the associations between DSL and mental health or wellbeing. In the regression models, DSL, major predisposing factors (age, gender, ethnic group, marital status) and SES (urban/rural residence, educational attainment, and relatively living standard) are controlled for in all the four models. Having any chronic diseases, ADL or IADL are only controlled for in Model 2 and Model 4 so that it is possible to examine how the associations between DSL and depression or life satisfaction will change.

**Table 5:** Impacts of DSL on mental health and wellbeing.

**All persons aged 60+**	**Depression**		**Life satisfaction**	
	**Model 1**	**Model 2**	**Model 3**	**Model 4**
DSL	0.785^*^	0.610^*^	−0.593^*^	−0.491^*^
Any chronic diseases		0.457^*^		−0.157
Any ADL limitation		0.776^*^		−0.667^*^
Any IADL limitation		0.777^*^		−0.253^*^
**60–64 (Omitted)**				
65–69	−0.021	−0.128	0.154	0.198
70–74	−0.057	−0.288^*^	0.262^*^	0.361^*^
75+	−0.483^*^	−0.868^*^	0.404^*^	0.619^*^
**Male (omitted)**				
Female	0.491^*^	0.349^*^	−0.246^*^	−0.131
Minority	0.106	−0.044	−0.097	0.009
Urban	−0.514^*^	−0.554^*^	0.047	0.103
**Primary schooling**				
Second schooling	−0.445^*^	−0.303^*^	0.411^*^	0.370^*^
College and above	−0.401	−0.331	0.637	0.376
Better				
Average	0.201	0.281	−0.345	−0.515
Worse	0.702^*^	0.659^*^	−1.430^*^	−1.528^*^
**Married with spouse present**				
Married with spouse away	−0.296	−0.299	−0.425	−0.518
Separated/Divorced/Widowed	0.371^*^	0.335^*^	−0.403^*^	−0.380^*^
Single	−0.098	−0.278	−0.701^*^	−0.204
Cons	−2.779	−2.441	3.479	3.414
R-square	0.069	0.099	−0.056	−0.060

* *Indicates significant at a significance level of 5%*.

*CHARLS 2013. http://charls.pku.edu.cn/en*.

After controlling for major predisposing factors and SES, the following results were evident: (1) DSL had significant influences on both mental health and wellbeing, however, the absolute value of association between DSL and depression (0.785) in Model 1 is higher than that of the association between DSL and life satisfaction (−0.593) in Model 3. (2) Older people who have any chronic diseases are more likely to report depression but there is no significant difference in their reporting of life satisfaction when compared to those without any chronic diseases. (3) Having any ADL or IADL limitation is significantly associated with both depression and less life satisfaction. (4) After controlling for having any ADL limitation, IADL limitation or any chronic diseases in Model 2 and Model 4, the estimated coefficients of DSL decreased significantly from 0.785 in Model 1 to 0.610 in Model 2 and from −0.593 in Model 3 to −0.491 in Model 4 respectively. This means that the observed associations between DSL and depression can be partially explained by having any chronic diseases, ADL limitation or IADL limitation, while the observed associations between DSL and life satisfaction can be partially explained by having any ADL limitation or IADL limitation, but not by having any chronic diseases.

## Discussion

DSL is prevalent in the older adult Chinese population with more than half (57.2%) of the CHARLS 2013 respondents reporting DSL. The prevalence rates of sensory loss in the current study are higher than Western population-based studies of vision and hearing loss, while are similar to other population-based studies of vision and hearing loss in China. For example, Swenor et al. (33) is a US study that reported the prevalence of DSL as 13.8% for adults aged 60 years and over. This prevalence difference may be attributed to the use of different methodologies; for example, self -report was used in the CHARLS data, whereas Swenor et al. (33) evaluated DSL on the basis of postautorefraction visual acuity and pure-tone audiometric measurements.

A further finding of this study [as also reported by Heine et al. (8)] was that the prevalence of DSL increased from 55.2% in 60–64 year olds to 58.5% in adults aged 75 years and over and that DSL is more prevalent in those living in rural areas, those with worse than the average living standard, or those without textural education. Detailed comparison of DSL prevalence in Chinese population studies can be found in Heine et al. (8).

Results of the current study also suggest that DSL impacted on depressive symptoms and life satisfaction. Correlation and regression analysis found that in comparison to people with no DSL, people with DSL reported difficulties completing ADLs and IADLs, more depressive symptoms and less life satisfaction. Correlations were all significant. Furthermore, results of regression analyses revealed that DSL has a higher impact on depression than on life satisfaction. However, these relationships are in part impacted by other variables. The impact of DSL on depression is influenced by the presence of any chronic illness and any ADL or IADL limitation. The impact of DSL on life satisfaction is influenced by the presence of any ADL or IADL limitation. Harada et al. (34) examined the vision and hearing acuity of 843 people aged 65 years and over living in a rural Japanese town and found similar findings. Based on a questionnaire about sensory acuity, depression, subjective poor health, and functional activity, Harada and colleagues found that sensory loss (or impairment) was significantly associated with negative wellbeing in older adults.

The estimated coefficients for other variables are all consistent with expectations. It was found that: (1) advanced age is significantly associated with less depression, as well as more life satisfaction, probably because the older cohort has more positive life attitudes; (2) females are more likely to report depression and less life satisfaction; (3) there is no significant difference in depression and life satisfaction between ethnic groups; (4) urban older people are less likely to report depression and there is no significant difference in life satisfaction between urban and rural older people; (5) older people with secondary schooling are less likely to report depression but more likely to report life satisfaction probably due to their relative low expectation when compared those with high education; (6) worse living standard is significantly associated with depression and less life satisfaction; (7) separated/ divorced/ widowed older people are more likely to report depression and less life satisfaction and; (8) single older people are more likely to report less life satisfaction, but not significant difference in reporting depression.

## Limitations

This study is an empirical study based on the most recently available survey data on health and retirement in China. Though the innovation of the design and findings of this study is limited, it is novel and valuable to investigate the impact of DSL on the quality of life in a rapidly aging society such as China. Our findings are timely due to the paucity of empirical papers on DSL in China.

Another limitation of the study is not exploring whether correctly-adjusted assistive devices (hearing aids, glasses) can significantly improve life satisfaction and mental health. Future research can investigate the difference between respondents who had the appropriate use of aids and the correctly adjusted/adapted aids to help with vision and hearing loss, vs. those who did not.

## Conclusions

DSL is thus a significant factor affecting the mental health and wellbeing of older adults in China through its relationships with IADL and ADL limitations and comorbidity with chronic illnesses. Many of the impacts of DSL including its impact on mental health and wellbeing may be ameliorated by appropriate rehabilitation and the use of aids (10). Since older adults with DSL frequently experience communication difficulty leading to poor psychosocial functioning and social isolation, rehabilitation options should include communication training for older people with DSL and their careers. Identifying and managing the personal, situational, and environmental factors that contribute to communication breakdown (14) are key to effective communication training programs. Furthermore, adequate use of visual and hearing aids may be of benefit. Useful visual aids include assistive technologies that can be adjusted for volume for people who have an additional hearing loss (such as a talking GPS system) or a hearing aid that adds volume. Assistive listening devices such as Remote Microphone technology (which overcomes the signal to noise ratio of a noisy environment) may be of particular benefit for those people with DSL. For people with DSL, overcoming communication difficulties positively impacts on socialization and health often leading to improved quality of life and wellbeing (14).

Health professionals, however, need to understand that older people with DSL may also have other chronic conditions that need to be addressed as part of a comprehensive treatment and rehabilitation program. Interventions need to address the functional aspects of DSL as well as the possible mental health impacts. This requires a cross-disciplinary collaboration across vision, hearing, mental health and chronic illness services in referral, assessment and rehabilitation (15). This is a common issue worldwide where many health systems fail to provide good integration of services across professions especially for older people with complex needs.

As discussed by Heine et al. (8) the availability and accessibility in China of specialty hearing and vision services for older people, particularly those with poor financial resources, is low. Whilst the Chinese primary health care reforms aim to deliver better care across the whole population there are gaps in the ability of the system to address the complex needs of older people with sensory losses, particularly those who have associated mental health conditions.

## Ethics Statement

The original CHARLS survey data was approved by the Ethical Review Committee of Peking University, and all participants signed informed consent at the time of participation. There is no need for ethics approval for second hand data users.

## Author Contributions

CH and CB conceptualized the paper. CG conducted all the statistical analyses. CH drafted the paper and CG and CB revised the paper. All authors have final approval of the published article and agree to be accountable for all aspects of the work in ensuring that questions related to the accuracy or integrity of any part of the work are appropriately investigated and resolved.

### Conflict of Interest Statement

The authors declare that the research was conducted in the absence of any commercial or financial relationships that could be construed as a potential conflict of interest.

## Footnotes

^1^As this paper focuses on DSL among older people, we did not review any literature about DSL and depression in younger people. DSL in young people is often diagnosed in early life and has a separate literature. The psychological response to deaf-blindness in early life is likely to be different to DSL acquired in later life as an age-related condition.
